# Supplementary material for: Patterns of beverage purchases amongst British households: A latent class analysis
Source: PLoS Med. 2020 Sep 8;17(9):e1003245. doi: 10.1371/journal.pmed.1003245 (PMC7478648; doi:10.1371/journal.pmed.1003245)
Supplement: S1 Appendix — (DOC) [file pmed.1003245.s001.doc]

**S1 Appendix - STROBE Statement**

|  | Item No | Recommendation |
| --- | --- | --- |
| **Title and abstract** | 1 | (*a*) Indicate the study’s design with a commonly used term in the title or the abstract  *The title describes the study design as “latent class analysis”. The abstract further specify that this is a “cross-sectional latent class analysis”.* |
|  |  | (*b*) Provide in the abstract an informative and balanced summary of what was done and what was found  *The abstract describes the methods and findings.* |
| Introduction | | |
| Background/rationale | 2 | Explain the scientific background and rationale for the investigation being reported  *The background and rationale are described in the Introduction, paragraphs 1-3.* |
| Objectives | 3 | State specific objectives, including any prespecified hypotheses.  *The specific aims of the study are stated in the Introduction, paragraph 4.* |
| Methods | | |
| Study design | 4 | Present key elements of study design early in the paper.  *The study design is discussed in paragraph 4 of the Introduction and paragraphs 2-14 of the Methods section* |
| Setting | 5 | Describe the setting, locations, and relevant dates, including periods of recruitment, exposure, follow-up, and data collection.  *The setting, location, and relevant dates, exposure, and data collection are discussed in paragraphs 2-7 of the Methods section* |
| Participants | 6 | (*a*) Give the eligibility criteria, and the sources and methods of selection of participants.  *Selection of the sample is discussed in paragraph 2 of the Methods section* |
|  |  |  |
| Variables | 7 | Clearly define all outcomes, exposures, predictors, potential confounders, and effect modifiers. Give diagnostic criteria, if applicable.  *All variables are described in the Measures subsection of the method section (paragraphs 4-7 of the Methods section)* |
| Data sources/ measurement | 8* | For each variable of interest, give sources of data and details of methods of assessment (measurement). Describe comparability of assessment methods if there is more than one group.  *All variables are described in the Measures subsection of the Methods section (paragraphs 4-7 of the Methods section)* |
| Bias | 9 | Describe any efforts to address potential sources of bias.  *The advantage of using purchase data compared to self-reported intake data is explained in paragraph 4 of the Introduction. Exclusion criteria to avoid bias due to mass purchasing are described in paragraph 3 of the Methods section. The use of relative measures instead of absolute measures is described in paragraph 6 of the Methods section* |
| Study size | 10 | Explain how the study size was arrived at  *The study sample size is described in paragraph 3 of the Methods section* |
| Quantitative variables | 11 | Explain how quantitative variables were handled in the analyses. If applicable, describe which groupings were chosen and why  *Use of variables is discussed in the Statistical Analysis subsection* |
| Statistical methods | 12 | (*a*) Describe all statistical methods, including those used to control for confounding *Statistical methods are discussed in the Statistical Analysis subsection* |
|  |  |  |
|  |  | (*c*) Explain how missing data were addressed  *Missing data handling is described in paragraph 8 of the Methods section* |
|  |  | (*d*) If applicable, explain how loss to follow-up was addressed  *Not applicable* |
|  |  | (*e*) Describe any sensitivity analyses  *Sensitivity analyses are described in the Sensitivity Analysis subsection* |
| Results | | |
| Participants | 13* | (a) Report numbers of individuals at each stage of study—eg numbers potentially eligible, examined for eligibility, confirmed eligible, included in the study, completing follow-up, and analysed  *Data process flow is described in a flowchart (Fig 1) and in paragraph 3 of the Methods section* |
|  |  | (b) Give reasons for non-participation at each stage  *Data process flow is described in a flowchart (Fig 1) and in paragraph 3 of the Methods section* |
|  |  | (c) Consider use of a flow diagram  *Data process flow is described in a flowchart (Fig 1)* |
| Descriptive data | 14* | (a) Give characteristics of study participants (eg demographic, clinical, social) and information on exposures and potential confounders  *Percentages/proportion are reported in Table 1* |
|  |  | (b) Indicate number of participants with missing data for each variable of interest *Number of participants with missing data for each variable of interest is reported in Table 1* |
|  |  |  |
| Outcome data | 15* | Report numbers of outcome events or summary measures over time  *Median (min-max) or mean(SD) are presented in Table 1* |
| Main results | 16 | (*a*) Give unadjusted estimates and, if applicable, confounder-adjusted estimates and their precision (eg, 95% confidence interval). Make clear which confounders were adjusted for and why they were included.  *Unadjusted estimates are presented in Table 2, multivariable results are presented in Tables 3 and 4.* |
|  |  | (*b*) Report category boundaries when continuous variables were categorized  *Min-Max values are presented for each tertile variable in Table 1* |
|  |  | (*c*) If relevant, consider translating estimates of relative risk into absolute risk for a meaningful time period  *Not relevant* |
| Other analyses | 17 | Report other analyses done—eg analyses of subgroups and interactions, and sensitivity analyses –  *Sensitivity analyses are presented in paragraph 12 of the Results section and in S5 Appendix* |
| Discussion | | |
| Key results | 18 | Summarise key results with reference to study objectives  *Results are summarised in paragraphs 1 of Discussion section* |
| Limitations | 19 | Discuss limitations of the study, taking into account sources of potential bias or imprecision. Discuss both direction and magnitude of any potential bias  *Limitations are discussed in paragraph 6 of the Discussion section (Limitations subsection)* |
| Interpretation | 20 | Give a cautious overall interpretation of results considering objectives, limitations, multiplicity of analyses, results from similar studies, and other relevant evidence *Final paragraph of Discussion section (Conclusion subsection).* |
| Generalisability | 21 | Discuss the generalisability (external validity) of the study results  *Paragraph 7 of the Discussion section* |
| Other information | | |
| Funding | 22 | Give the source of funding and the role of the funders for the present study and, if applicable, for the original study on which the present article is based.  *Metadata* |

*Give information separately for exposed and unexposed groups.

**Note:** An Explanation and Elaboration article discusses each checklist item and gives methodological background and published examples of transparent reporting. The STROBE checklist is best used in conjunction with this article (freely available on the Web sites of PLoS Medicine at http://www.plosmedicine.org/, Annals of Internal Medicine at http://www.annals.org/, and Epidemiology at http://www.epidem.com/). Information on the STROBE Initiative is available at http://www.strobe-statement.org.
