# Supplementary material for: Patterns of beverage purchases amongst British households: A latent class analysis
Source: PLoS Med. 2020 Sep 8;17(9):e1003245. doi: 10.1371/journal.pmed.1003245 (PMC7478648; doi:10.1371/journal.pmed.1003245)
Supplement: S2 Appendix — (DOCX) [file pmed.1003245.s002.docx]

**S2 Appendix - Food and beverage group definitions**

|  | **Description** |
| --- | --- |
| **Beverages** |  |
| Sugar sweetened beverages (SSBs) | Sugary beverages, juices or squashes not included in the diet beverage category.  We assumed a dilution rate of 1:4 as used by the British Soft Drinks Association for concentrated squashes |
| Diet beverages | Beverages branded as ‘diet’, ‘low sugar’ or ‘No added sugar’, or with less than 1g of sugar/100ml;  Juices branded as ‘diet’, ‘low sugar’ or ‘No added sugar’, or with less than 1g of sugar/100ml;  Squashes branded as ‘diet’, ‘low sugar’ or ‘No added sugar’ and with less than 3g of sugar/100ml;  Flavoured water with less than 1g sugar/100mL  We assumed a dilution rate of 1:4 as used by the British Soft Drinks Association for concentrated squashes |
| Fruit juices & milk-based beverages | 100% fruit juices and milk-based beverages (e.g. flavoured milk, chocolate milk), excluding pure milk |
| Beer and cider | Any beer or cider |
| Wine | Wine, including sparkling wine and fortified wine |
| Bottled water | Still or sparkling bottled water |
| **Food** |  |
| Fruits and vegetables | Fresh and frozen fruits; fresh frozen and tinned vegetables, excluding potatoes |
| Sweet snacks | Confectionary, chewing gum, chocolate (plain and filled); sweet biscuits, cereal and fruit bars; desserts, puddings, cakes, home baking, dried fruits |
| **Less healthy food and beverages** | Food products scoring above 4 points and drinks scoring above 1 point on the UK Department of Health and Social Care Nutrient profiling model. The nutrient profiling model assigns points to products based on the content of energy, sugar, saturated fat, salt, NSP fibre, protein and fruit and vegetables. |
