# Supplementary material for: Patterns of beverage purchases amongst British households: A latent class analysis
Source: PLoS Med. 2020 Sep 8;17(9):e1003245. doi: 10.1371/journal.pmed.1003245 (PMC7478648; doi:10.1371/journal.pmed.1003245)
Supplement: S3 Appendix — (DOCX) [file pmed.1003245.s003.docx]

**S3 Appendix - Model comparison of the latent class analysis**

**Table S3 – Model comparison of the latent class analysis**

| No. of classes | AIC | BIC | VLMR - LRT | BLRT | Entropy | Sample Proportion (%) per Class Based on Most Likely Class Membership |
| --- | --- | --- | --- | --- | --- | --- |
| 1 | 114389 | 114474 | - | - | 1.000 | 100 |
| 2 | 113016 | 113192 | <0.001 | <0.001 | 0.676 | 59/41 |
| 3 | 112545 | 112814 | <0.001 | <0.001 | 0.604 | 56/14/30 |
| 4 ^a^ | 112295 | 112656 | <0.001 | <0.001 | 0.523 | 14/17/41/28 |
| 5 ^a^ | 112182 | 112635 | 0.001 | <0.001 | 0.555 | 4/18/38/24/16 |
| 6 ^a^ | 112001 | 112545 | <0.001 | <0.001 | 0.621 | 39/18/5/13/18/7 |
| **7 ^b^** | **111898** | **112535** | **0.004** | **<0.001** | **0.708** | **18/16/6/7/18/4/30** |
| 8 ^b^ | 111827 | 112555 | 0.005 | <0.001 | 0.692 | 18/31/7/7/5/3/14/15 |
| 9 ^b^ | 111760 | 112580 | 0.024 | <0.001 | 0.668 | 8/22/15/5/7/9/4/11/19 |
| 10 ^c^ | 111741 | 112653 | 1.000 | <0.001 | 0.665 | 7/5/24/11/19/6/16/1/7/3 |

AIC = Akaike information criterion; BIC = Bayesian information criterion; VLMR - LRT = Vuong-Lo-Mendell-Rubin likelihood ratio test; BLRT = bootstrap likelihood ratio test

^a^ Number of random starts increased to 800 with 80 optimization phases.

^b^ Number of random starts increased to 10000 with 2000 optimization phases.

^c^ Number of random starts increased to 20000 with 4000 optimization phases.

**Figure S3 – BIC by number of latent classes**
