# Supplementary material for: Patterns of beverage purchases amongst British households: A latent class analysis
Source: PLoS Med. 2020 Sep 8;17(9):e1003245. doi: 10.1371/journal.pmed.1003245 (PMC7478648; doi:10.1371/journal.pmed.1003245)
Supplement: S4 Appendix — (DOCX) [file pmed.1003245.s004.docx]

**S4 Appendix – Classification of beverage purchasing into seven latent classes using 8,675 British households, that reported regular beverage purchases between 4th January 2016 and 1st January 2017 (52 weeks)**

| **Volume tertile of beverage category** | **Class 1:**  **SSB** | **Class 2:**  **Diet** | **Class 3:**  **Fruit & Milk** | **Class 4:**  **Beer & Cider** | **Class 5:**  **Wine** | **Class 6:**  **Water** | **Class 7:**  **Diverse** |
| --- | --- | --- | --- | --- | --- | --- | --- |
| SSBs |  |  |  |  |  |  |  |
| Low | 0.000 | 0.536 | 0.336 | 0.522 | 0.569 | 0.480 | 0.214 |
| Middle | 0.080 | 0.407 | 0.5 | 0.324 | 0.290 | 0.520 | 0.418 |
| High | 0.920 | 0.057 | 0.163 | 0.154 | 0.142 | 0.000 | 0.369 |
| Diet beverages |  |  |  |  |  |  |  |
| Low | 0.379 | 0.000 | 0.594 | 0.236 | 0.410 | 0.605 | 0.366 |
| Middle | 0.327 | 0.321 | 0.292 | 0.362 | 0.313 | 0.316 | 0.365 |
| High | 0.294 | 0.679 | 0.114 | 0.402 | 0.277 | 0.078 | 0.269 |
| Fruit juices/milk-based beverages |  |  |  |  |  |  |  |
| Low | 0.257 | 0.492 | 0.000 | 0.587 | 0.523 | 0.501 | 0.165 |
| Middle | 0.405 | 0.320 | 0.000 | 0.279 | 0.295 | 0.499 | 0.409 |
| High | 0.338 | 0.189 | 1.000 | 0.135 | 0.182 | 0.000 | 0.426 |
| Beer and cider |  |  |  |  |  |  |  |
| Low | 0.605 | 0.582 | 0.666 | 0.000 | 0.227 | 0.640 | 0.000 |
| Middle | 0.271 | 0.418 | 0.276 | 0.000 | 0.262 | 0.259 | 0.495 |
| High | 0.124 | 0.000 | 0.058 | 1.000 | 0.512 | 0.102 | 0.505 |
| Wine |  |  |  |  |  |  |  |
| Low | 0.671 | 0.581 | 0.484 | 0.457 | 0.000 | 0.616 | 0.028 |
| Middle | 0.283 | 0.403 | 0.311 | 0.543 | 0.000 | 0.293 | 0.502 |
| High | 0.045 | 0.016 | 0.205 | 0.000 | 1.000 | 0.091 | 0.470 |
| Bottled water |  |  |  |  |  |  |  |
| Low | 0.388 | 0.400 | 0.353 | 0.484 | 0.358 | 0.006 | 0.251 |
| Middle | 0.290 | 0.393 | 0.263 | 0.354 | 0.350 | 0.000 | 0.379 |
| High | 0.323 | 0.207 | 0.383 | 0.161 | 0.291 | 0.994 | 0.370 |
| Most likely class membership, n (%) | 1,543 (18%) | 1,402 (16%) | 529 (6%) | 644 (7%) | 1,586 (18%) | 352 (4%) | 2,619 (30%) |

*Note:* Values are probability of being in the volume tertile of the beverage category, unless otherwise noted.

SSB, sugar-sweetened beverage.
