# Supplementary material for: Patterns of beverage purchases amongst British households: A latent class analysis
Source: PLoS Med. 2020 Sep 8;17(9):e1003245. doi: 10.1371/journal.pmed.1003245 (PMC7478648; doi:10.1371/journal.pmed.1003245)
Supplement: S5 Appendix — (DOCX) [file pmed.1003245.s005.docx]

**S5 Appendix – Sensitivity analyses**

Results from sensitive analyses are presented for:

1) 7-class model of the restricted sample (N=7,446)

2) 6-class model of the restricted sample (N=7,446)

3) 6-class model of the main analytical sample (N=8,675), using quintile beverage indicators

Part 1: Box-plots


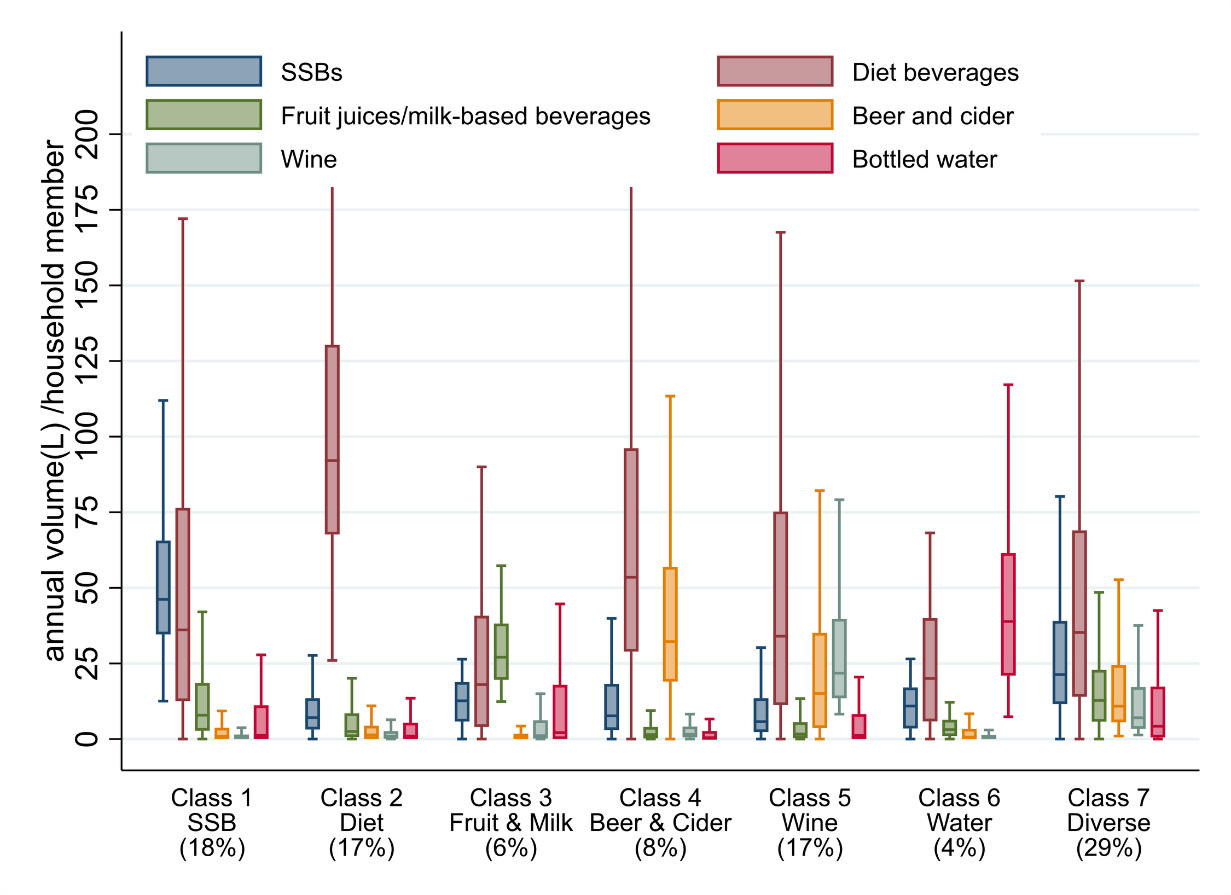


Figure S5.1 – Box-plot of the beverage categories by latent class for the 7-class model (N=7,446)

^
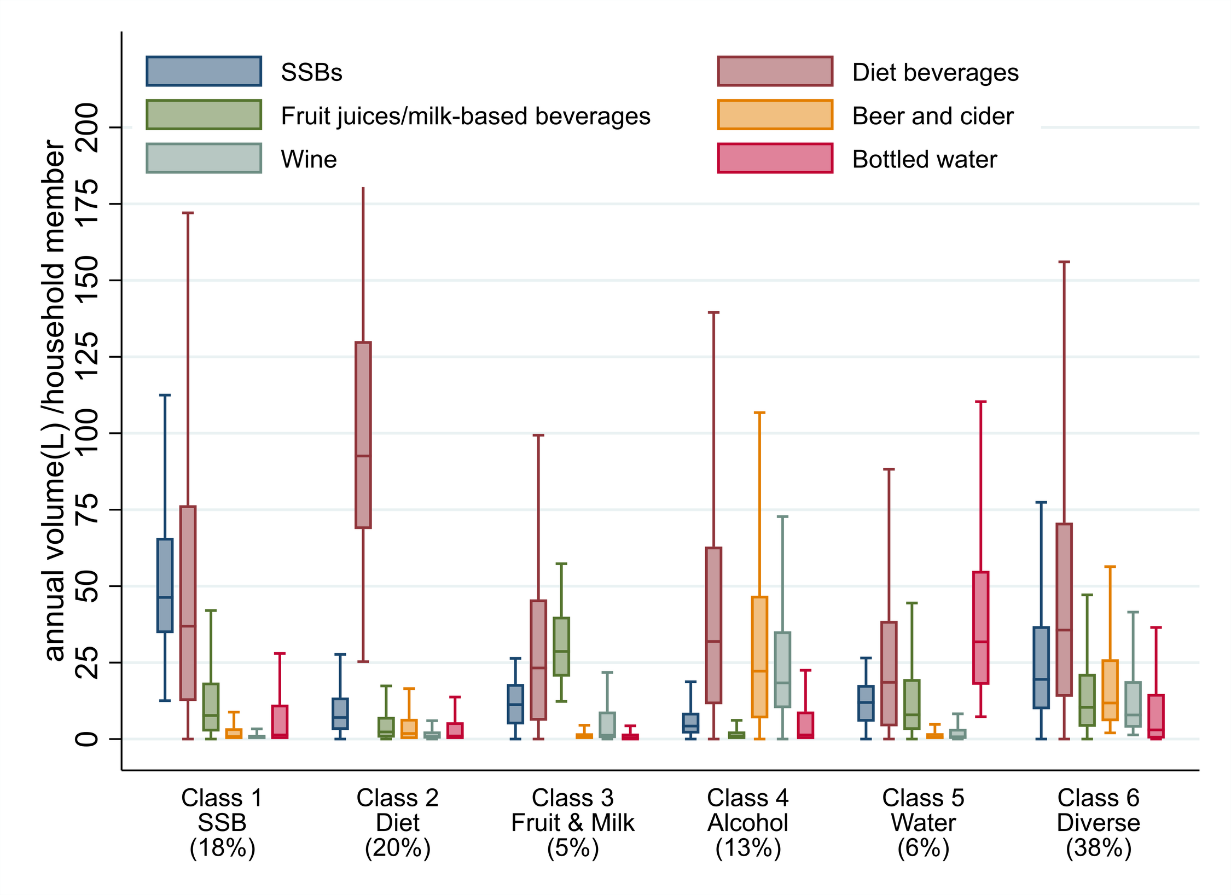
^

Figure S5.2 – Box-plot of the beverage categories by latent class for the 6-class model (N=7,446)
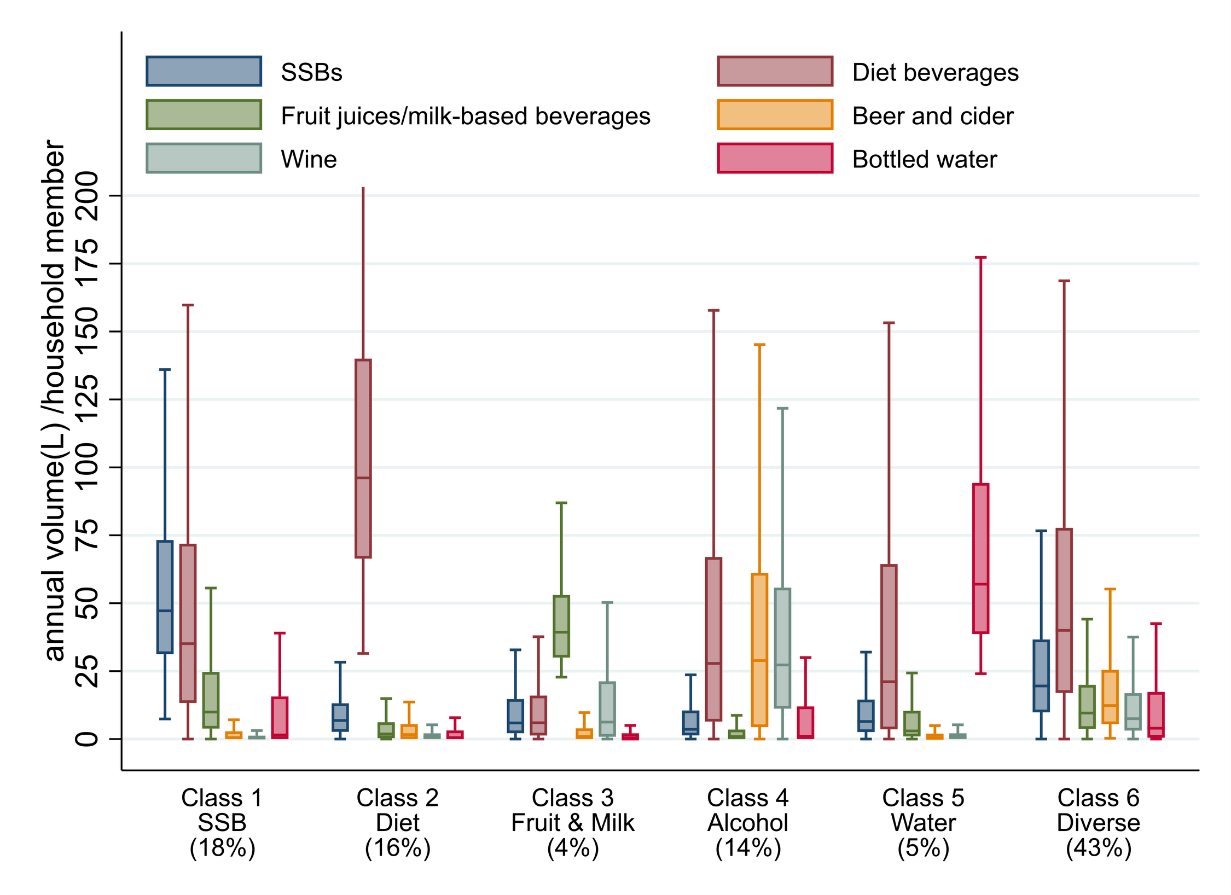


Figure S5.3 – Box-plot of the beverage categories by latent class for the 6-class model based on quintile beverage indicators (N=8,675)

Part 2: Associations with socio-demographic variables and BMI status

Table S5.1 - Relative risk ratios (SE, p-value) of membership in specified class compared to “Diverse” Class in multinomial logistic regression

|  |  | 7-class | model | (N=7446) | 6-class | model | (N=7446) | 6-class | model ^a^ | (N=8765) |
| --- | --- | --- | --- | --- | --- | --- | --- | --- | --- | --- |
|  |  | RRR | SE | p-value | RRR | SE | p-value | RRR | SE | p-value |
|  |  | **Class 1 "SSB"** | |  | **Class 1 "SSB"** | |  | **Class 1** | **"SSB”** |  |
| Region | Midland | 0.967 | 0.233 | 0.884 | 0.728 | 0.216 | 0.141 | 0.747 | 0.203 | 0.151 |
| (Ref: London) | North East | 0.751 | 0.331 | 0.386 | 0.547 | 0.293 | 0.040 | 0.756 | 0.283 | 0.322 |
|  | Yorkshire | 0.735 | 0.231 | 0.182 | 0.548 | 0.212 | 0.005 | 0.553 | 0.213 | 0.005 |
|  | Lancashire | 0.682 | 0.274 | 0.162 | 0.548 | 0.252 | 0.017 | 0.435 | 0.237 | <0.001 |
|  | South | 0.830 | 0.241 | 0.439 | 0.688 | 0.223 | 0.093 | 0.664 | 0.217 | 0.059 |
|  | Scotland | 0.890 | 0.250 | 0.642 | 0.694 | 0.230 | 0.112 | 0.663 | 0.226 | 0.070 |
|  | East England | 0.803 | 0.269 | 0.414 | 0.649 | 0.248 | 0.081 | 0.726 | 0.230 | 0.164 |
|  | Wales and West | 0.698 | 0.291 | 0.216 | 0.505 | 0.254 | 0.007 | 0.381 | 0.275 | <0.001 |
|  | South West | 0.419 | 0.353 | 0.014 | 0.305 | 0.346 | 0.001 | 0.522 | 0.333 | 0.051 |
| Income | £20,000 - 29,999 | 0.727 | 0.187 | 0.087 | 0.643 | 0.165 | 0.007 | 0.727 | 0.150 | 0.033 |
| (Ref: <£20,000) | £30,000 - 39,999 | 0.611 | 0.204 | 0.016 | 0.456 | 0.185 | <0.001 | 0.515 | 0.178 | <0.001 |
|  | £40,000 - 49,999 | 0.320 | 0.242 | <0.001 | 0.283 | 0.222 | <0.001 | 0.244 | 0.250 | <0.001 |
|  | ≥£50,000 | 0.239 | 0.247 | <0.001 | 0.204 | 0.226 | <0.001 | 0.190 | 0.244 | <0.001 |
| Life stage | Young family (0-4 yrs) | 2.333 | 0.301 | 0.005 | 2.208 | 0.281 | 0.005 | 1.817 | 0.278 | 0.032 |
| (Ref: pre-family) | Middle family (5-9 yrs) | 1.186 | 0.301 | 0.569 | 1.188 | 0.282 | 0.543 | 1.005 | 0.285 | 0.987 |
|  | Older family (10+ yrs) | 1.292 | 0.269 | 0.341 | 1.426 | 0.253 | 0.162 | 1.735 | 0.254 | 0.030 |
|  | Older dependents | 1.598 | 0.277 | 0.091 | 1.556 | 0.254 | 0.082 | 0.899 | 0.260 | 0.682 |
|  | Empty nesters | 1.484 | 0.259 | 0.127 | 1.151 | 0.237 | 0.553 | 1.091 | 0.241 | 0.719 |
|  | Retired | 1.419 | 0.281 | 0.214 | 0.969 | 0.244 | 0.899 | 0.985 | 0.255 | 0.953 |
| Occupational social grade | C1 | 1.288 | 0.204 | 0.214 | 1.236 | 0.182 | 0.243 | 1.087 | 0.196 | 0.672 |
| (Ref: A&B) | C2 | 1.408 | 0.238 | 0.150 | 1.351 | 0.212 | 0.156 | 1.110 | 0.224 | 0.643 |
|  | D | 1.677 | 0.251 | 0.039 | 1.684 | 0.227 | 0.022 | 1.687 | 0.226 | 0.021 |
|  | E | 10.719 | 0.669 | <0.001 | 9.098 | 0.542 | <0.001 | 4.154 | 0.294 | <0.001 |
| BMI category | Underweight | 4.272 | 1.063 | 0.172 | 2.075 | 0.504 | 0.148 | 2.138 | 0.484 | 0.117 |
| (Ref: Normal) | Overweight | 1.009 | 0.152 | 0.954 | 0.973 | 0.137 | 0.843 | 1.023 | 0.136 | 0.867 |
|  | Obese | 1.033 | 0.163 | 0.842 | 1.017 | 0.149 | 0.907 | 1.097 | 0.146 | 0.524 |
|  |  | **Class 2 "Diet"** | |  | **Class 2 "Diet"** | |  | **Class 2 "Diet"** | |  |
| Region | Midland | 1.415 | 0.260 | 0.182 | 1.405 | 0.259 | 0.188 | 1.311 | 0.238 | 0.255 |
| (Ref: London) | North East | 1.372 | 0.348 | 0.365 | 1.570 | 0.319 | 0.158 | 1.586 | 0.311 | 0.138 |
|  | Yorkshire | 1.432 | 0.253 | 0.157 | 1.252 | 0.256 | 0.379 | 1.206 | 0.246 | 0.448 |
|  | Lancashire | 1.848 | 0.274 | 0.025 | 1.650 | 0.278 | 0.072 | 1.143 | 0.253 | 0.595 |
|  | South | 1.297 | 0.267 | 0.329 | 1.255 | 0.268 | 0.397 | 1.169 | 0.255 | 0.539 |
|  | Scotland | 1.582 | 0.267 | 0.086 | 1.336 | 0.277 | 0.294 | 1.468 | 0.256 | 0.134 |
|  | East England | 1.547 | 0.282 | 0.123 | 1.536 | 0.280 | 0.126 | 1.394 | 0.265 | 0.210 |
|  | Wales and West | 1.330 | 0.306 | 0.351 | 1.158 | 0.288 | 0.608 | 1.432 | 0.261 | 0.168 |
|  | South West | 0.901 | 0.354 | 0.769 | 0.828 | 0.359 | 0.598 | 1.080 | 0.353 | 0.826 |
| Income | £20,000 - 29,999 | 0.931 | 0.196 | 0.713 | 0.749 | 0.182 | 0.112 | 0.773 | 0.169 | 0.126 |
| (Ref: <£20,000) | £30,000 - 39,999 | 0.858 | 0.210 | 0.464 | 0.628 | 0.196 | 0.018 | 0.731 | 0.185 | 0.089 |
|  | £40,000 - 49,999 | 0.614 | 0.231 | 0.035 | 0.536 | 0.227 | 0.006 | 0.456 | 0.221 | <0.001 |
|  | ≥£50,000 | 0.516 | 0.229 | 0.004 | 0.463 | 0.220 | <0.001 | 0.450 | 0.213 | <0.001 |
| Life stage | Young family (0-4 yrs) | 1.565 | 0.287 | 0.118 | 1.887 | 0.272 | 0.020 | 1.416 | 0.252 | 0.167 |
| (Ref: pre-family) | Middle family (5-9 yrs) | 1.183 | 0.274 | 0.539 | 1.564 | 0.259 | 0.085 | 1.115 | 0.244 | 0.654 |
|  | Older family (10+ yrs) | 0.649 | 0.268 | 0.106 | 0.733 | 0.260 | 0.233 | 0.546 | 0.262 | 0.021 |
|  | Older dependents | 0.962 | 0.263 | 0.883 | 0.907 | 0.253 | 0.699 | 0.533 | 0.241 | 0.009 |
|  | Empty nesters | 1.133 | 0.243 | 0.606 | 0.963 | 0.230 | 0.868 | 0.764 | 0.220 | 0.222 |
|  | Retired | 1.042 | 0.269 | 0.879 | 0.645 | 0.246 | 0.075 | 0.765 | 0.235 | 0.254 |
| Occupational social grade | C1 | 0.842 | 0.180 | 0.339 | 0.899 | 0.172 | 0.541 | 0.809 | 0.172 | 0.218 |
| (Ref: A&B) | C2 | 0.961 | 0.213 | 0.849 | 1.064 | 0.200 | 0.758 | 1.047 | 0.193 | 0.812 |
|  | D | 0.913 | 0.242 | 0.707 | 1.126 | 0.231 | 0.608 | 0.907 | 0.227 | 0.666 |
|  | E | 5.624 | 0.659 | 0.009 | 5.646 | 0.538 | 0.001 | 2.050 | 0.299 | 0.017 |
| BMI category | Underweight | 1.984 | 1.121 | 0.541 | 1.349 | 0.610 | 0.624 | 1.368 | 0.605 | 0.605 |
| (Ref: Normal) | Overweight | 1.174 | 0.158 | 0.311 | 1.204 | 0.152 | 0.222 | 1.239 | 0.153 | 0.162 |
|  | Obese | 1.954 | 0.160 | <0.001 | 2.335 | 0.152 | <0.001 | 2.366 | 0.149 | <0.001 |
|  |  | **Class 3 "Fruit &** | | **Milk"** | **Class 3 "Fruit &** | | **Milk"** | **Class 3 "Fruit & Milk"** | | |
| Region | Midland | 0.411 | 0.331 | 0.007 | 0.516 | 0.397 | 0.096 | 0.564 | 0.364 | 0.115 |
| (Ref: London) | North East | 0.248 | 0.635 | 0.028 | 0.311 | 0.655 | 0.075 | 0.264 | 1.014 | 0.189 |
|  | Yorkshire | 0.252 | 0.377 | <0.001 | 0.360 | 0.376 | 0.007 | 0.529 | 0.370 | 0.085 |
|  | Lancashire | 0.491 | 0.348 | 0.041 | 0.735 | 0.385 | 0.423 | 0.364 | 0.448 | 0.024 |
|  | South | 0.364 | 0.335 | 0.003 | 0.341 | 0.398 | 0.007 | 0.353 | 0.430 | 0.015 |
|  | Scotland | 0.552 | 0.312 | 0.056 | 0.542 | 0.353 | 0.082 | 0.598 | 0.354 | 0.146 |
|  | East England | 0.680 | 0.334 | 0.248 | 0.859 | 0.372 | 0.684 | 0.681 | 0.410 | 0.350 |
|  | Wales and West | 0.473 | 0.383 | 0.050 | 0.358 | 0.473 | 0.030 | 0.656 | 0.394 | 0.285 |
|  | South West | 0.013 | 5.826 | 0.454 | 0.039 | 3.522 | 0.356 | 0.213 | 0.972 | 0.112 |
| Income | £20,000 - 29,999 | 0.668 | 0.263 | 0.125 | 0.523 | 0.283 | 0.022 | 0.666 | 0.281 | 0.149 |
| (Ref: <£20,000) | £30,000 - 39,999 | 0.441 | 0.308 | 0.008 | 0.317 | 0.354 | 0.001 | 0.600 | 0.344 | 0.138 |
|  | £40,000 - 49,999 | 0.371 | 0.354 | 0.005 | 0.340 | 0.431 | 0.012 | 0.368 | 0.530 | 0.059 |
|  | ≥£50,000 | 0.414 | 0.321 | 0.006 | 0.408 | 0.374 | 0.017 | 0.727 | 0.385 | 0.406 |
| Life stage | Young family (0-4 yrs) | 1.029 | 0.474 | 0.951 | 1.338 | 0.633 | 0.645 | 1.505 | 1.198 | 0.732 |
| (Ref: pre-family) | Middle family (5-9 yrs) | 1.195 | 0.423 | 0.673 | 1.480 | 0.589 | 0.505 | 1.279 | 1.232 | 0.842 |
|  | Older family (10+ yrs) | 0.927 | 0.400 | 0.850 | 1.241 | 0.568 | 0.704 | - | - | - |
|  | Older dependents | 1.283 | 0.417 | 0.550 | 1.254 | 0.589 | 0.702 | 0.906 | 1.333 | 0.941 |
|  | Empty nesters | 0.930 | 0.389 | 0.852 | 1.171 | 0.534 | 0.768 | 4.807 | 1.069 | 0.142 |
|  | Retired | 2.646 | 0.378 | 0.010 | 3.501 | 0.499 | 0.012 | 19.767 | 1.052 | 0.005 |
| Occupational social grade | C1 | 0.722 | 0.241 | 0.175 | 0.681 | 0.274 | 0.161 | 0.614 | 0.279 | 0.081 |
| (Ref: A&B) | C2 | 0.598 | 0.311 | 0.098 | 0.740 | 0.319 | 0.345 | 0.355 | 0.377 | 0.006 |
|  | D | 0.396 | 0.385 | 0.016 | 0.410 | 0.460 | 0.053 | 0.121 | 0.860 | 0.014 |
|  | E | 1.828 | 0.807 | 0.455 | 1.079 | 0.870 | 0.930 | 0.682 | 0.446 | 0.391 |
| BMI category | Underweight | 1.900 | 1.439 | 0.655 | - | - | - | 0.663 | 1.166 | 0.724 |
| (Ref: Normal) | Overweight | 0.864 | 0.208 | 0.481 | 1.015 | 0.227 | 0.947 | 0.444 | 0.247 | 0.001 |
|  | Obese | 0.691 | 0.245 | 0.131 | 0.604 | 0.305 | 0.099 | 0.378 | 0.284 | 0.001 |
|  |  | **Class 4 "Beer & Cider"** | | |  |  |  |  |  |  |
| Region | Midland | 3.053 | 0.464 | 0.016 |  |  |  |  |  |  |
| (Ref: London) | North East | 4.536 | 0.523 | 0.004 |  |  |  |  |  |  |
|  | Yorkshire | 2.812 | 0.457 | 0.024 |  |  |  |  |  |  |
|  | Lancashire | 3.350 | 0.480 | 0.012 |  |  |  |  |  |  |
|  | South | 0.979 | 0.577 | 0.971 |  |  |  |  |  |  |
|  | Scotland | 1.502 | 0.536 | 0.449 |  |  |  |  |  |  |
|  | East England | 2.740 | 0.504 | 0.046 |  |  |  |  |  |  |
|  | Wales and West | 2.748 | 0.501 | 0.043 |  |  |  |  |  |  |
|  | South West | 1.254 | 0.638 | 0.723 |  |  |  |  |  |  |
| Income | £20,000 - 29,999 | 1.010 | 0.266 | 0.970 |  |  |  |  |  |  |
| (Ref: <£20,000) | £30,000 - 39,999 | 0.935 | 0.301 | 0.825 |  |  |  |  |  |  |
|  | £40,000 - 49,999 | 0.661 | 0.353 | 0.241 |  |  |  |  |  |  |
|  | ≥£50,000 | 0.728 | 0.348 | 0.361 |  |  |  |  |  |  |
| Life stage | Young family (0-4 yrs) | 2.201 | 0.440 | 0.073 |  |  |  |  |  |  |
| (Ref: pre-family) | Middle family (5-9 yrs) | 0.849 | 0.483 | 0.734 |  |  |  |  |  |  |
|  | Older family (10+ yrs) | 0.423 | 0.521 | 0.098 |  |  |  |  |  |  |
|  | Older dependents | 1.219 | 0.422 | 0.638 |  |  |  |  |  |  |
|  | Empty nesters | 1.848 | 0.391 | 0.116 |  |  |  |  |  |  |
|  | Retired | 1.861 | 0.414 | 0.134 |  |  |  |  |  |  |
| Occupational social grade | C1 | 1.114 | 0.311 | 0.729 |  |  |  |  |  |  |
| (Ref: A&B) | C2 | 2.259 | 0.327 | 0.013 |  |  |  |  |  |  |
|  | D | 1.837 | 0.391 | 0.120 |  |  |  |  |  |  |
|  | E | 5.783 | 0.766 | 0.022 |  |  |  |  |  |  |
| BMI category | Underweight | 5.888 | 1.237 | 0.152 |  |  |  |  |  |  |
| (Ref: Normal) | Overweight | 1.045 | 0.238 | 0.853 |  |  |  |  |  |  |
|  | Obese | 1.293 | 0.245 | 0.294 |  |  |  |  |  |  |
|  |  | **Class 5 "Wine"** | |  | **Class 4 "Alcohol"** | |  | **Class 4 "Alcohol"** | |  |
| Region | Midland | 1.883 | 0.333 | 0.057 | 1.323 | 0.348 | 0.420 | 1.207 | 0.282 | 0.505 |
| (Ref: London) | North East | 2.951 | 0.419 | 0.010 | 1.770 | 0.402 | 0.155 | 2.219 | 0.346 | 0.021 |
|  | Yorkshire | 1.594 | 0.319 | 0.144 | 0.877 | 0.338 | 0.698 | 1.439 | 0.268 | 0.175 |
|  | Lancashire | 3.108 | 0.337 | 0.001 | 2.155 | 0.340 | 0.024 | 1.659 | 0.285 | 0.076 |
|  | South | 1.381 | 0.339 | 0.341 | 0.732 | 0.381 | 0.412 | 0.995 | 0.287 | 0.987 |
|  | Scotland | 1.557 | 0.335 | 0.186 | 0.841 | 0.354 | 0.625 | 1.141 | 0.288 | 0.646 |
|  | East England | 1.791 | 0.359 | 0.104 | 1.177 | 0.389 | 0.676 | 1.262 | 0.310 | 0.452 |
|  | Wales and West | 2.537 | 0.368 | 0.011 | 1.026 | 0.388 | 0.948 | 1.439 | 0.303 | 0.229 |
|  | South West | 1.323 | 0.469 | 0.550 | 1.134 | 0.446 | 0.778 | 1.412 | 0.377 | 0.359 |
| Income | £20,000 - 29,999 | 1.045 | 0.239 | 0.853 | 0.838 | 0.237 | 0.455 | 1.050 | 0.184 | 0.792 |
| (Ref: <£20,000) | £30,000 - 39,999 | 1.373 | 0.249 | 0.203 | 0.733 | 0.266 | 0.242 | 1.040 | 0.204 | 0.848 |
|  | £40,000 - 49,999 | 0.589 | 0.327 | 0.105 | 0.428 | 0.343 | 0.013 | 0.547 | 0.274 | 0.028 |
|  | ≥£50,000 | 1.044 | 0.288 | 0.881 | 0.666 | 0.312 | 0.192 | 0.783 | 0.256 | 0.340 |
| Life stage | Young family (0-4 yrs) | 0.515 | 0.792 | 0.403 | 0.661 | 0.771 | 0.592 | 0.169 | 0.987 | 0.072 |
| (Ref: pre-family) | Middle family (5-9 yrs) | 0.855 | 0.610 | 0.797 | 0.558 | 0.758 | 0.441 | 0.332 | 0.571 | 0.053 |
|  | Older family (10+ yrs) | 0.085 | 2.260 | 0.276 | 0.146 | 1.547 | 0.213 | - | - | - |
|  | Older dependents | 1.994 | 0.498 | 0.166 | 1.813 | 0.498 | 0.232 | 0.645 | 0.377 | 0.244 |
|  | Empty nesters | 5.983 | 0.453 | <0.001 | 4.141 | 0.446 | 0.001 | 3.497 | 0.303 | <0.001 |
|  | Retired | 7.877 | 0.470 | <0.001 | 3.518 | 0.448 | 0.005 | 4.125 | 0.313 | <0.001 |
| Occupational social grade | C1 | 0.836 | 0.227 | 0.431 | 0.649 | 0.239 | 0.070 | 0.696 | 0.192 | 0.059 |
| (Ref: A&B) | C2 | 0.783 | 0.276 | 0.375 | 0.727 | 0.296 | 0.280 | 0.710 | 0.230 | 0.135 |
|  | D | 0.747 | 0.302 | 0.333 | 0.773 | 0.307 | 0.400 | 0.667 | 0.252 | 0.108 |
|  | E | 1.768 | 0.752 | 0.449 | 1.759 | 0.641 | 0.378 | 0.834 | 0.343 | 0.597 |
| BMI category | Underweight | 2.284 | 1.305 | 0.527 | 1.164 | 0.730 | 0.835 | 1.251 | 0.686 | 0.744 |
| (Ref: Normal) | Overweight | 0.820 | 0.188 | 0.294 | 0.968 | 0.195 | 0.866 | 0.789 | 0.158 | 0.134 |
|  | Obese | 0.631 | 0.207 | 0.026 | 0.705 | 0.225 | 0.119 | 0.625 | 0.174 | 0.007 |
|  |  |  |  |  |  |  |  |  |  |  |
|  |  | **Class 6 "Water"** | |  | **Class 5 "Water"** | |  | **Class 5 "Water"** | |  |
| Region | Midland | 0.337 | 0.386 | 0.005 | 0.222 | 0.429 | <0.001 | 0.271 | 0.382 | 0.001 |
| (Ref: London) | North East | 0.256 | 0.711 | 0.055 | 0.190 | 0.620 | 0.007 | 0.210 | 0.646 | 0.016 |
|  | Yorkshire | 0.223 | 0.453 | 0.001 | 0.182 | 0.405 | <0.001 | 0.289 | 0.372 | 0.001 |
|  | Lancashire | 0.441 | 0.414 | 0.048 | 0.253 | 0.438 | 0.002 | 0.596 | 0.289 | 0.073 |
|  | South | 0.640 | 0.324 | 0.168 | 0.519 | 0.295 | 0.026 | 0.607 | 0.290 | 0.085 |
|  | Scotland | 0.250 | 0.513 | 0.007 | 0.406 | 0.339 | 0.008 | 0.365 | 0.369 | 0.006 |
|  | East England | 0.402 | 0.456 | 0.046 | 0.285 | 0.428 | 0.003 | 0.450 | 0.371 | 0.031 |
|  | Wales and West | 0.482 | 0.420 | 0.083 | 0.444 | 0.346 | 0.019 | 0.447 | 0.372 | 0.030 |
|  | South West | 0.236 | 0.629 | 0.022 | 0.156 | 0.696 | 0.008 | 0.343 | 0.546 | 0.050 |
| Income | £20,000 - 29,999 | 0.910 | 0.314 | 0.765 | 0.763 | 0.282 | 0.335 | 0.902 | 0.250 | 0.681 |
| (Ref: <£20,000) | £30,000 - 39,999 | 0.717 | 0.355 | 0.349 | 0.461 | 0.318 | 0.015 | 0.619 | 0.292 | 0.101 |
|  | £40,000 - 49,999 | 0.457 | 0.408 | 0.054 | 0.436 | 0.357 | 0.020 | 0.412 | 0.364 | 0.015 |
|  | ≥£50,000 | 0.330 | 0.380 | 0.004 | 0.354 | 0.335 | 0.002 | 0.488 | 0.313 | 0.022 |
| Life stage | Young family (0-4 yrs) | 1.179 | 0.470 | 0.725 | 1.270 | 0.387 | 0.537 | 0.672 | 0.410 | 0.332 |
| (Ref: pre-family) | Middle family (5-9 yrs) | 0.964 | 0.430 | 0.931 | 1.000 | 0.374 | 1.000 | 0.672 | 0.377 | 0.293 |
|  | Older family (10+ yrs) | 0.528 | 0.445 | 0.151 | 0.653 | 0.363 | 0.241 | 0.382 | 0.435 | 0.027 |
|  | Older dependents | 0.845 | 0.422 | 0.690 | 0.872 | 0.348 | 0.694 | 0.544 | 0.372 | 0.101 |
|  | Empty nesters | 0.897 | 0.381 | 0.775 | 0.519 | 0.342 | 0.055 | 0.875 | 0.309 | 0.666 |
|  | Retired | 1.005 | 0.405 | 0.990 | 0.496 | 0.366 | 0.055 | 0.974 | 0.316 | 0.935 |
| Occupational social grade | C1 | 0.454 | 0.290 | 0.007 | 0.566 | 0.252 | 0.023 | 0.546 | 0.240 | 0.012 |
| (Ref: A&B) | C2 | 0.811 | 0.342 | 0.540 | 0.524 | 0.348 | 0.063 | 0.517 | 0.308 | 0.032 |
|  | D | 0.706 | 0.384 | 0.365 | 0.547 | 0.368 | 0.101 | 0.400 | 0.361 | 0.011 |
|  | E | 2.713 | 0.808 | 0.217 | 2.428 | 0.646 | 0.170 | 1.228 | 0.404 | 0.611 |
| BMI category | Underweight | 2.889 | 1.272 | 0.404 | 1.423 | 0.796 | 0.658 | 0.899 | 1.096 | 0.923 |
| (Ref: Normal) | Overweight | 0.803 | 0.252 | 0.385 | 0.710 | 0.237 | 0.148 | 0.834 | 0.220 | 0.410 |
|  | Obese | 0.911 | 0.269 | 0.729 | 1.076 | 0.233 | 0.753 | 1.174 | 0.214 | 0.454 |

Data are from GB Kantar FMCG

Note: A&B - higher and intermediate managerial, administrative or professional occupations; C1- supervisory, clerical and junior managerial administrative or professional occupations ; C2 - skilled manual workers; D- semi- or unskilled manual workers; E - state pensioners, casual or lowest grade workers, and those unemployed with state benefits.

^a^ Model based on quintile beverage indicators.

Part 3: Associations with socio-demographic variables

Table S5.2 - Mean (SE) energy and nutrient content of purchases by latent class

|  | 7-class model (N=7446) | | 6-class model (N=7446) | |
| --- | --- | --- | --- | --- |
| Variable | Mean | SE | Mean | SE |
|  | **Class 1 "SSB"** | | **Class 1 "SSB"** | |
| Total (kcal/household member/day) ^a^ | 1875.377 | 20.91 | 1876.782 | 20.187 |
| % from sweet snacks | 18.47 | 0.245 | 18.314 | 0.234 |
| % from fruits and vegetables | 6.182 | 0.118 | 6.228 | 0.113 |
| % from less healthy food and beverages ^b^ | 54.364 | 0.284 | 54.096 | 0.273 |
| Fat (g/1000kcal) | 40.124 | 0.173 | 40.178 | 0.167 |
| Saturated Fat (g/1000kcal) | 15.644 | 0.089 | 15.664 | 0.086 |
| Protein (g/1000kcal) | 32.449 | 0.18 | 32.433 | 0.173 |
| Carbohydrates (g/1000kcal) | 123.077 | 0.419 | 122.611 | 0.41 |
| Sugar (g/1000kcal) | 62.415 | 0.392 | 62.255 | 0.378 |
| NSP Fibre (g/1000kcal) | 8.285 | 0.065 | 8.283 | 0.062 |
| Sodium (g/1000kcal) | 0.99 | 0.006 | 0.987 | 0.005 |
|  | **Class 2 "Diet"** | | **Class 2 "Diet"** | |
| Total (kcal/household member/day) ^a^ | 1703.487 | 21.253 | 1727.746 | 22.891 |
| % from sweet snacks | 18.72 | 0.268 | 18.918 | 0.288 |
| % from fruits and vegetables | 7.928 | 0.146 | 7.754 | 0.156 |
| % from less healthy food and beverages ^b^ | 51.171 | 0.296 | 51.538 | 0.324 |
| Fat (g/1000kcal) | 39.914 | 0.193 | 39.873 | 0.208 |
| Saturated Fat (g/1000kcal) | 15.52 | 0.095 | 15.459 | 0.103 |
| Protein (g/1000kcal) | 35.714 | 0.211 | 35.787 | 0.226 |
| Carbohydrates (g/1000kcal) | 119.503 | 0.473 | 120.411 | 0.519 |
| Sugar (g/1000kcal) | 53.978 | 0.438 | 54.134 | 0.474 |
| NSP Fibre (g/1000kcal) | 9.471 | 0.076 | 9.439 | 0.082 |
| Sodium (g/1000kcal) | 1.094 | 0.006 | 1.105 | 0.007 |
|  | **Class 3 "Fruit & Milk"** | | **Class 3 "Fruit & Milk"** | |
| Total (kcal/household member/day) ^a^ | 1627.849 | 32.202 | 1718.371 | 42.992 |
| % from sweet snacks | 17.067 | 0.456 | 16.869 | 0.555 |
| % from fruits and vegetables | 8.621 | 0.262 | 8.314 | 0.323 |
| % from less healthy food and beverages ^b^ | 48.711 | 0.518 | 48.413 | 0.682 |
| Fat (g/1000kcal) | 39.791 | 0.33 | 39.455 | 0.431 |
| Saturated Fat (g/1000kcal) | 15.813 | 0.172 | 15.867 | 0.22 |
| Protein (g/1000kcal) | 33.891 | 0.446 | 33.598 | 0.68 |
| Carbohydrates (g/1000kcal) | 121.499 | 0.833 | 121.12 | 1.128 |
| Sugar (g/1000kcal) | 62.929 | 0.703 | 62.295 | 0.902 |
| NSP Fibre (g/1000kcal) | 9.521 | 0.135 | 9.441 | 0.173 |
| Sodium (g/1000kcal) | 0.936 | 0.011 | 0.909 | 0.016 |
|  | **Class 4 "Beer & Cider"** | | |  |
| Total (kcal/household member/day) ^a^ | 1713.799 | 35.735 |  |  |
| % from sweet snacks | 15.255 | 0.4 |  |  |
| % from fruits and vegetables | 7.235 | 0.226 |  |  |
| % from less healthy food and beverages ^b^ | 48.848 | 0.508 |  |  |
| Fat (g/1000kcal) | 39.853 | 0.314 |  |  |
| Saturated Fat (g/1000kcal) | 15.073 | 0.152 |  |  |
| Protein (g/1000kcal) | 35.552 | 0.343 |  |  |
| Carbohydrates (g/1000kcal) | 112.496 | 0.779 |  |  |
| Sugar (g/1000kcal) | 49.231 | 0.7 |  |  |
| NSP Fibre (g/1000kcal) | 8.943 | 0.121 |  |  |
| Sodium (g/1000kcal) | 1.087 | 0.01 |  |  |
|  | **Class 5 "Wine"** | | **Class 4 "Alcohol"** | |
| Total (kcal/household member/day) ^a^ | 1477.295 | 86.533 | 1405.03 | 59.331 |
| % from sweet snacks | 10.914 | 1.122 | 10.896 | 0.771 |
| % from fruits and vegetables | 8.64 | 0.313 | 8.544 | 0.254 |
| % from less healthy food and beverages ^b^ | 41.703 | 1.76 | 42.202 | 1.071 |
| Fat (g/1000kcal) | 38.524 | 0.586 | 38.633 | 0.433 |
| Saturated Fat (g/1000kcal) | 14.333 | 0.353 | 14.312 | 0.245 |
| Protein (g/1000kcal) | 36.644 | 0.757 | 36.467 | 0.492 |
| Carbohydrates (g/1000kcal) | 100.542 | 2.638 | 101.339 | 1.685 |
| Sugar (g/1000kcal) | 40.989 | 2.928 | 41.945 | 1.613 |
| NSP Fibre (g/1000kcal) | 9.398 | 0.163 | 9.361 | 0.139 |
| Sodium (g/1000kcal) | 1.065 | 0.019 | 1.066 | 0.013 |
|  | **Class 6 "Water"** | | **Class 5 "Water"** | |
| Total (kcal/household member/day) ^a^ | 1526.443 | 41.899 | 1489.337 | 37.973 |
| % from sweet snacks | 16.496 | 0.549 | 17.33 | 0.534 |
| % from fruits and vegetables | 9.148 | 0.356 | 9.374 | 0.336 |
| % from less healthy food and beverages ^b^ | 49.346 | 0.685 | 49.619 | 0.634 |
| Fat (g/1000kcal) | 41.23 | 0.436 | 41.147 | 0.388 |
| Saturated Fat (g/1000kcal) | 15.753 | 0.237 | 15.853 | 0.215 |
| Protein (g/1000kcal) | 35.114 | 0.432 | 35.015 | 0.405 |
| Carbohydrates (g/1000kcal) | 118.105 | 0.979 | 118.951 | 0.924 |
| Sugar (g/1000kcal) | 55.498 | 0.871 | 58.132 | 0.786 |
| NSP Fibre (g/1000kcal) | 9.962 | 0.183 | 9.981 | 0.172 |
| Sodium (g/1000kcal) | 0.98 | 0.015 | 0.982 | 0.013 |
|  | **Class 7 "Diverse"** | | **Class 6 "Diverse"** | |
| Total (kcal/household member/day) ^a^ | 1861.986 | 23.685 | 1859.597 | 19.546 |
| % from sweet snacks | 15.599 | 0.246 | 15.482 | 0.209 |
| % from fruits and vegetables | 7.483 | 0.142 | 7.529 | 0.12 |
| % from less healthy food and beverages ^b^ | 50.573 | 0.307 | 50.322 | 0.262 |
| Fat (g/1000kcal) | 40.258 | 0.195 | 40.256 | 0.163 |
| Saturated Fat (g/1000kcal) | 15.691 | 0.102 | 15.679 | 0.086 |
| Protein (g/1000kcal) | 33.375 | 0.202 | 33.552 | 0.172 |
| Carbohydrates (g/1000kcal) | 114.807 | 0.506 | 113.902 | 0.431 |
| Sugar (g/1000kcal) | 56.348 | 0.425 | 55.354 | 0.363 |
| NSP Fibre (g/1000kcal) | 8.786 | 0.074 | 8.821 | 0.062 |
| Sodium (g/1000kcal) | 0.998 | 0.006 | 0.997 | 0.005 |

Data are from GB Kantar FMCG

*Note:* each energy/nutrient variable was treated as an auxiliary variable and means (SE) were estimated using the manual 3-Step BCH method, adjusting for household size and number of children to account for unequal purchases due to household composition.

The model based on quintile beverage indicators did not converge.

^a^ Value under-reported by around 3% on average. In addition, some puddings, biscuits, and bread products, as well as all bacon and sausages, slimming products and milkshake mixes were excluded because of inconsistent nutrient information reported at product level. Products excluded could account for up 130kcal per household member per day.

^b^ Defined using the UK Department of Health and Social Care nutrient profiling model.

NSP, non-starch polysaccharides
