# Supplementary material for: Patterns of beverage purchases amongst British households: A latent class analysis
Source: PLoS Med. 2020 Sep 8;17(9):e1003245. doi: 10.1371/journal.pmed.1003245 (PMC7478648; doi:10.1371/journal.pmed.1003245)
Supplement: Attachment — Submitted filename: Response 1 to reviewers.docx [file pmed.1003245.s006.docx]

**Authors’ response**

*We would like to thank the reviewers and the editors for their positive and constructive feedback. We revised the manuscript and adopted the vast majority of the changes suggested by the reviewers. We also adopted the changes requested by the editors. We believe that the changes further clarify our argument.*

Requests from the editors:

Abstract
Methods and findings- please mention briefly the source of income and occupation social grade data. If this is available at FMCG please mention this. Please also mention the threshold of “regular purchases” and “high volumes of SSB”.

*Response: The abstract (pp 3-4) was edited accordingly. Reference to “high volumes of SSB” was removed*

Conclusions
* Please address the study implications without overreaching what can be concluded from the data; the phrase "In this study, we observed ..." may be useful.

*Response: The conclusions were edited accordingly (p4)*

* Please interpret the study based on the results presented in the abstract, emphasizing what is new without overstating your conclusions.

*Response: The conclusions were edited accordingly (p4)*

* Please avoid vague statements such as "these results have major implications for policy/clinical care". Mention only specific implications substantiated by the results.

*Response: The manuscript was edited accordingly*

* Please avoid assertions of primacy ("We report for the first time....")

*Response: The manuscript was edited accordingly*

At this stage, we ask that you include a short, non-technical Author Summary of your research to make findings accessible to a wide audience that includes both scientists and non-scientists. The Author Summary should immediately follow the Abstract in your revised manuscript. This text is subject to editorial change and should be distinct from the scientific abstract. Please see our author guidelines for more information: [https://journals.plos.org/plosmedicine/s/revising-your-manuscript#loc-author-summary](https://protect-eu.mimecast.com/s/clspCB6q0Uq2P8hEkXfs?domain=journals.plos.org)

*Response: the author summary is now added (p5)*

Methods
It might help to state which data were obtained from the FMCG early in the methods section

*Response: We now clearly state that demographic data are also provided by Kantar (pp7-8 subsection study sample)*

Is there an alternative phrase for “empty nesters” ? Not only colloquial, I do wonder if this type of label could be perceived as stigmatising

*Response: The marketing label “empty nesters” was defined by Kantar. Reviewer 2 has similar comments about the variable label. These are defined by Kantar. Following reviewer 2’s suggestion we kept it as such, for consistency purposes. We have nonetheless noted in the revised version of the manuscript that these labels were defined by Kantar (p9 last paragraph)*

It would be helpful to define thresholds for beverage classes like “low wine” or “low diet” briefly. This information may be in the supplementary files but it would be beneficial in the main text for clarity.

*Response: to avoid confusion, this terminology was removed following reviewer comments.*

Please ensure that the study is reported according to the STROBE guideline, and include the completed STROBE checklist as Supporting Information. When completing the checklist, please use section and paragraph numbers, rather than page numbers. Please add the following statement, or similar, to the Methods: "This study is reported as per the Strengthening the Reporting of Observational Studies in Epidemiology STROBE guideline (S1 Checklist)."

Please report your study according to the relevant guideline, which can be found here: [http://www.equator-network.org/](https://protect-eu.mimecast.com/s/d3m4CD8vnCpGj3HESIb3?domain=equator-network.org)

*Response: the STROBE checklist was completed and the study reported according to relevant guideline (S1 Appendix)*

Results
Please provide 95% confidence intervals along with all p-values. In addition, please provide exact p-values unless p<0.001

*Response: We have edited the manuscript accordingly, and included p-values when relevant. (throughout)*

Discussion
You note that BMI was self reported in the limitations section but I didn’t see this in the methods. Could you please provide this information there?

*Response: We have edited the manuscript accordingly (p9 last paragraph)*

Please use UK or Great Britain consistently in the manuscript

*Response: We have edited the manuscript accordingly and are now using GB consistently in the manuscript.*

Please clarify if the FMCG is representative of the UK population. Please also clarify if the channel islands are included in the panel

*Response: Kantar FMCG panel is representative of the Great Britain population. Great Britain does not include the Channel Islands, nor Northern Island. We define Great Britain specifically as England, Wales and Scotland in the methods section (p7 last paragraph).*

Comments from the reviewers:

**Reviewer #1:** This is a statistical review of manuscript PMEDICINE-D-20-00344_R1. The manuscript reads well. I have two minor comments.

Minor comments:
* Abstract: you state ""… restricted our analyses to consumers who purchase beverages regularly (n=6,339)." I know that it is very clearly defined in the Methods, but perhaps define what you mean regularly in the Abstract for the reader who may only read the abstract.

*Response: Thank you for the suggestion. This information is now added to the abstract (p3 paragraph 2).*

* Page 8: "complete cases were therefore analysed". How many were in the final analysis? I think that this is a crucial point to make. One would want to know how many (in absolute and relative terms) records out of the 6,339 have been used for the LCA.

*Response: Information (in absolute and relative terms) is now added in the main text:*

*“complete cases were therefore analysed (n=6,432 out of 8,675; 74%)” (p10 paragraph2)*

*[Note that the sample size of the main analysis increased slightly following changes carried out based on reviewers’ comments]*

**Reviewer #2:** Thank you for the opportunity to review this interesting study. This study used a latent class analysis to characterise patterns of beverage purchases using data from the 2016 UK Kantar consumer panel. The authors identified 7 patterns with regard to beverage purchasing and related household and panel member characteristics to these patterns. I think the paper has implications for public policy, and the use of the consumer panel data is unique, although not without limitations (these are mostly discussed by the authors in the paper). My specific comments and suggestions are as follows:

1. You state that my "My study does not require an ethics statement." However, this study does involve human participants. In a recent study in PLOS Medicine (Taillie et al., 2020;17:e1003015) the authors have included a statement under methods that the study was exempt from review from their relevant review board as it uses secondary, de-identified data. Could you please check with your ethics committee to confirm that the current study does not need ethical review?

*Response: Similar to a paper published in PLOS Medicine based on the same dataset in the UK (Ejlerskov et al. , 2018 e:1002712), ethical approval was not required for this study. A statement has now been added in the methods section to clarify this (p8 last paragraph).*

2. Pg 7. When you state that "For each household, we computed overall mean daily energy and nutrients purchased per household member from all food and beverages.", were adults and children treated equally, or was some allowance made for the fact that children in a household might have different energy/nutrient requirements and not eat as many of the purchased energy and nutrients?

*Response: As in Scheelbeek et al 2019 (doi:10.1136/bmj.l4786), adults and children were treated equally.*

*Energy requirements do vary depending on age, gender and activity levels. For example, according to US guidelines (*[*https://health.gov/dietaryguidelines/2015/guidelines/*](https://health.gov/dietaryguidelines/2015/guidelines/)*), an active 16 year-old boy would require 3200kcal, an inactive 60+ women 1600kcal and a 2 year-old 1000kcal. In this study, we only know the exact age and gender of the main respondent. Furthermore, we do not know the activity level of any of the household members for accurate adjustment. As a result, mean daily values reported per household member represent a rough estimations of how much households purchase on average per member, keeping in mind that these values should not be compared against the reference intake values (e.g. 2000kcal for energy). This contributes to explaining why the reported absolute values might seem ‘low’ compared to dietary intake values reported from dietary surveys.*

*The absence of adjustment is now clearly stated. We added “without adjustment for age or sex”.*

3. Pg 7. When you refer to "selected food groups of public health concern", I found this wording ambiguous, I initially thought you meant you were interested in less healthy food and beverages which included the sub-groups of sweet snacks and fruit and vegetables. This is obviously not what you meant, so perhaps if you state there were 3 food groups of interest this might help. I suggest using the word "interest" instead of "concern" as concern has negative connotations and fruit and vegetables are 'healthy'. Also, can you please clarify whether 'less healthy' food and beverages and 'sweet snacks' are mutually exclusive food groups? Typically in nutrition papers food groups are mutually exclusive, and if there is some overlap here it would be useful to make that clear to the reader.

*Response: Thank you for the suggestion. We replaced “concern” by “interest” (p9 paragraph 2). We also clearly indicated in the revised version of the manuscript that sweet snacks is a subset of the ‘less healthy’ category (p9 paragraph 2)*

4. I am interested in your rationale for mostly using % of purchased energy from food groups or nutrient density (e.g. in Table 5) although I note in Table 2 you present actual energy from purchased beverages. For studies that use dietary assessment of intakes the results are typically presented as % of energy or adjusted for energy intake to reduce measurement error (assuming that most participants misreport foods and beverages in the same direction and to a similar extent). However, this is not the case here as you are looking at purchases, and the consumer panel does not rely on self-reported intakes. On the other hand, I think it might help with the fact that the household composition differs, and houses have varying numbers of adults and children present.

*Response: Thank you for this comment. There are two main issues with absolute values in the Kantar data:*

*1) ages of all households members is unknown. Therefore, we could not adjust values for age and gender in the energy/nutrient calculation, so that absolute values are under-estimated (cf. comment #2 above);*

*2) non-systematic reporting of all purchases. This non-systematic reporting could not be properly accounted for when analyses are conducted at household level (as done in this paper), because the weights provided by Kantar are meant to be applied to products, not to households (see below extended answer to comment #6 of reviewer 2). Note however that contrary to dietary intake survey, we observe in general less bias towards the under-reporting of less healthy products (e.g. savoury snacks and sweet snacks), it is more that values are generally lower. In a previous paper, we have estimated total energy purchased per capita for take home purchases (Berger et al. 2019* [*http://dx.doi.org/10.1136/bmjnph-2019-000036*](http://dx.doi.org/10.1136/bmjnph-2019-000036)*). Annual values for 2016 were 1860.7 kcal, whereas in this paper the sample mean reported in Table 1 was 1798.2 kcal. So the values presented in this paper are likely to be under-reported by around 3% on average.*

*As a result, absolute differences in nutrients and energy are not fully meaningful. We presented total energy in order to have an idea of what type of households purchase more energy for at home consumption (adjusting for household size and number of children).*

*In the revised version of the manuscript, we clarified why we used relative measures as opposed to absolute measures (p9 paragraph 2). To avoid confusion, we have removed absolute values (except for the total energy) from the main tables. If the reviewer believes that the analysis of absolute values would be useful regardless we could add this to the supplementary material.*

5. Are the life stages named by Kantar? I assume they are but wonder if a couple who doesn't intend to have children would appreciate being labelled 'pre-family'. If they are labelled by Kantar I don't think you should change the names. Please make it clear in the text and perhaps in a footnote in Table 1 that the age range is (presumably) the age range of children in the household. What happens if a couple has two children aged 3 and 7? Would they be classed as young or middle family?

*Response: Indeed life stages are defined and labelled by Kantar. We have now made it clear in the text and better defined the variable. Essentially, the life stage is defined based on the age of the youngest child if the household has children or the age of the main respondent if not (pp9-10).*

6. I note that you start with 32,110 households but after exclusions you end up with only 6,339 households. About 20,000 households appear to be excluded because they have fewer than 14 days of purchasing in any quarter. What is the rationale for applying this criteria? My concern is that you are making assumptions about the typical way for people to shop. To be included in your final sample, each household would have to do more than one shop a week, some households may only do a weekly shop or may even do a fort-nightly or monthly shop. I think you should only exclude households where the expenditure on food and beverages is implausible - I think that Kantar have already done this (as mentioned in the discussion) and my view is that the very strict criteria you have additionally imposed is unnecessary and could result in a biased sample. Similarly, what is the rationale for excluding households that purchased less than 1L of beverages per week per household member?

*Response: We thank the reviewer for raising this very important question. A key issue with the Kantar data is that some participants do not report data consistently throughout the year. The panel, as designed by Kantar, does not primarily aim to be used for longitudinal household analysis. Instead, it provides time-series of purchases of certain goods, for different groups of people. The weights provided by Kantar allow to ensure that these time-series are representative. However, when we perform longitudinal household-level analysis, as it is the case in this study, we are unable to use these weights which are not only designed for households but also for products, and therefore we are unable to account for intermittent reporting at household-level. As a result, the only way to account for intermittent reporting is to exclude households who do not systematically report purchases throughout the study period. Rachel Griffith et al. at the Institute for Fiscal Studies (cited in the manuscript) have previously identified a threshold of number of days per quarter which seems to define regular reporters, on the basis of their knowledge of household purchasing frequency habits. This threshold is likely to be conservative as noted by the reviewer, although it should be stressed that any purchase reported on a given day would count (e.g. a bottle of water or a pack of crisps purchased at the corner store would count as one day of reporting, whereas this is generally not considered as ‘shopping’). Kantar themselves estimate that the average number of grocery trips per households is 17 trips per month (*[*https://www.kantarworldpanel.com/en/PR/Grocery-growth-slows-and-habits-change-as-nation-adapts*](https://www.kantarworldpanel.com/en/PR/Grocery-growth-slows-and-habits-change-as-nation-adapts)*).*

*Our preference was to be conservative and to follow what was previously done in relevant literature. This means that we ended up with a well-defined sample of regular purchasers, as opposed to being less strict and potentially introducing uncontrolled biases which we could not account for, and therefore having data that could not be interpretable. On a computational side, having a smaller number of observations was a corollary advantage. For example, in preliminary analyses we performed analyses on a broader sample, but convergence could not be obtained for the more complex models. This is a known problem with mixture models in general.*

*The rationale for excluding households that purchased 52L or less of beverage per year per household member was to have a better defined study population of regular beverage purchasers, who are those who should be the focus of policies targeting beverage consumption. Understanding the behaviours of irregular purchasers is less interesting from a policy perspective, because their low level of consumption means that the policy is unlikely to have any noticeable health benefit for them. Furthermore, in the data analysis, irregular purchasers are likely to introduce a lot of noise (due to many zeros or very small values), which is likely to be well captured by the LCA. Excluding them allowed to have a better-focused picture of policy-relevant purchase patterns.*

*We have edited the revised version of the manuscript to clarify the above, in particular, we have added the following text p8 paragraph 2:*

*“*We restricted our analyses to households who consistently participated to the panel and reported purchases during the year (i.e. >14 days of purchases in every quarter) to discard data from households with intermittent participation to the panel [24], and to avoid seasonality bias. We further restricted our analyses to households who regularly purchased beverages (>52L per year per household member) because they are more likely to be the target and to benefit from policies addressing unhealthy beverage intake.”

7. Flow chart. S1 Appendix. In the last box, I think it would be clearer if you changed "no more than" to "less than or equal to".

*Response: Thank you. This was changed as suggested (Fig1)*

8. Income. On page 7, you state the categories for household income. In Table 1 the same categories are labelled "Annual income of the main householder". Is it household income or income of the main panel member? Please check tables and text and make sure they are consistent and correct.

*Response: Thank you. This is now corrected in Table 1. Data are for household income.*

9. Table 1. It would be helpful if you included a footnote to describe the occupational social grade of the main shopper.

*Response: Thank you. This was added as suggested.*

10. Table 1. Under beverages purchased I suggest changing "total" to "all" or "all households" as total implies the associated value is a sum, but this is a median of all households.

*Response: Thank you. This was changed as suggested.*

11. Labels of the 7 latent classes. There doesn't appear to be consistency in the way the 7 latent classes are labelled. For example, for Class 2 (juices, low diet and water) the probability of being in the lowest tertile of diet beverage purchasing is 0.65. For class 7 (water) the same probability is 0.64, but this is not labelled "water, low diet and low alcohol". I appreciate the advantages of using simple labels, but then class 2 could just be called "juices" if class 7 is "water". Also, class 6 is labelled "low calories" but for every other class 'low calories' is referred to as 'diet' and the use of 'low' for class 6 is confusing because for all other classes 'low' means less of something, and class 6 is characterised by high purchasing of diet drinks.

*Response: Thank you. We have simplified the labels to make them more consistent as suggested. We now characterise latent class labels using what households most likely purchase (as opposed to not purchase).*

12. Pg 11. The sentence beginning "Conversely, households in class "Alcohol, low SSBs & juices…" - I think it should say 'energy' not 'food'.

*Response: Thank you. This is now corrected.*

13. Table 5. The total energy purchased per household member appears to be quite low, and indicates a substantial (3/4?) of food supply into each household is missing. In the discussion you note that 25-39% of food and beverages out-of-home purchases, but it seems there is still some purchases missing. Do you have any thoughts on this discrepancy?

*Response: In a previous paper, we have estimated total energy purchased per capita for take home purchases (Berger et al. 2019* [*http://dx.doi.org/10.1136/bmjnph-2019-000036*](http://dx.doi.org/10.1136/bmjnph-2019-000036)*). Annual values for 2016 were 1860.7 kcal, whereas in this paper the sample mean reported in Table 1 was 1798.2 kcal (this is slightly increased estimate due to the changes to sample criteria done during the revisions as suggested by reviewer 3). Note that both figures exclude 5% of purchases which could account for up to 130kcal per person per day. The difference between the two figures is likely due to the fact that we could not account for under-reporting in this study (see comment #4). So, these values are likely to be under-reported by around 3% on average.*

*The best comparison we can make is to NDNS data which shows 2015-2016 mean/median food intake to be 1860/1806 kcal for adults 19-64 years old (slightly less for children and elderly) which includes foods consumed both in and out of homes. NDNS data are however known to suffer from under reporting. Nonetheless, the similarity of our estimates (at-home purchases that might include waste) to overall intake means that it is unlikely that we would be missing ¾ of food supply.*

*Note that in Table 4 results are adjusted for household number and number of children, and presented at sample means (household size of 2.5 and a number of children of 0.42).*

*It should also be noted that the out of home purchases account for 25-39% of expenditure. How this translates to energy content consumed out-of-home remains unknown because of lack of data. Out of home food and beverages are more expensive, but can still be more energy-dense too. The lack of out-of-home data is a limitation of this study (and currently all studies using purchase data), as highlighted in the limitation section.*

*The reason for using relative measures and the problem of under-reporting are now further clarified in the revised version of the manuscript (p9 paragraph 2) and in the Tables.*

14. At the end of Table 2 you have absolute energy from purchased beverages. In Table 5 you present percentage of energy from purchased food groups. I wonder if you could present absolute energy and percentage of energy from purchased beverages and food groups. I think the absolute energy purchased is interesting, but also in your discussion and conclusion you state that a greater proportion of purchased energy comes from sweet snacks when compared to SSB but I don't think you have explicitly shown this in the results, although I think that it is true.

*Response: As discussed above, absolute values are under-estimated (by around 3% on average), which makes small differences more difficult to estimate. As a result, we only present total energy (absolute value) and present relative values in both Tables 1 and 4.*

*We have edited the sentence about the comparison between energy from SSBs and sweet snacks, as it was confusing (the discussion and conclusion were substantially edited).*

15. 1st paragraph of the discussion. You state that in Classes 1 and 3, purchasing of energy is higher than other classes; however, I don't think they have higher purchasing of energy than the diverse class.

*Response: The sentence was revised based on the new analyses conducted. We ensure that the sentence is now accurate. The class high “SSB” now has highest point estimate for total energy.*

16. Discussion, pg 12. When you say "healthier baskets" are you referring to shopping baskets/trolleys?

*Response: Yes. This is now clarified.*

**Reviewer #3:**

Summary
Thank you for the opportunity to review PLOS Medicine D-20-00344R1, "Pattern of beverage purchases amongst British households: A latent class analysis." This manuscript applied latent class analysis (LCA) to identify groups of beverage purchasers using data from about 6,000 households participating in the UK Kantar household purchase panel. Main strengths of the paper include the objectively-collected, longitudinal purchase data and consideration of multiple aspects of purchase behavior (e.g., beverage purchases, nutrient purchases per 1000 kcal, etc.).

In my read, the paper has two key limitations.

1. The paper would benefit from additional explanation on why LCA is the appropriate method for addressing the research question.
It was not clear how your approach and results uniquely inform policymaking in the UK or elsewhere. I appreciated the Abstract's statement that we need additional research to understand whom will be most affected by SSB policies. However, it was not clear to me how LCA helps advance this understanding. Why not examine demographic characteristics of high SSB consumers - why does an LCA need to be conducted? If the full pattern of purchases is of interest, why not examine cross-price elasticities across key categories of products? The justification for LCA in relation to policymaking was not clear to me.

*Response: We thank the reviewer for the suggestion to strengthen the rationale for using LCA to answer the questions addressed in this paper. The introduction (paragraphs 3-4), abstract, methods (p10 ‘statistical analysis’ section, 1^st^ paragraph)) and discussion (p26 paragraph 2) were modified to better highlight the usefulness of the approach. Essentially, LCA are useful to identify individuals or households at higher risk, which could then be the target of policies.*

*In our view, LCA can advance policymaking in two ways:*

*1) It can inform on gaps in the coverage of target population by current or proposed policies. In the UK context for example, the policy focusing on SSB price and reformulation, misses out households with higher risk of overweight/obesity who mostly consume diet beverages. These households would not have been specifically identified by simply looking at population average descriptive statistics (i.e. those purchasing high volumes of diet beverages for example).*

*2) LCA tells us about likely substitutes and therefore gives further indication on how groups adequately targeted by policies are likely to be affected. For example, price increase of SSBs could lead to substitution towards alcohol in the ‘diverse’ class (who purchase these) but much less likely in the ‘SSB’ class whose alcohol purchases are minimal. In the UK policy context, there are two mechanisms at play: product reformulation and behavioural change (induced by price increase and social marketing campaigns). Depending on the combination of purchases in the groups identified, these mechanisms may have differential effectiveness.*

*Note that in previous work, we have also investigated cross-price elasticities across key beverage groups. Cross-price elasticities are useful to understand the likely average effects of fiscal policies. However, they are limited in their ability to capture heterogeneities within the population subgroups by which they are usually calculated (e.g. BMI category or income). Elasticities become very difficult to calculate for more specific combinations of subgroups. LCA allows for better understanding of heterogeneity. For example, it was found that an increase in SSB price might increase volume of diet drinks, in general (cf. Quirmbach et al. 2018 doi:10.1136/jech-2017-209791). The present analysis however indicates that there are different types of households purchasing SSBs and diet beverages in different combinations (and volumes), which might in turn have different price responsiveness. This type of information is typically missing from demand systems and further missing from modelling studies. A next step following this piece of research might be to use these identified LC to calculate elasticities and have a better understanding of how price change might differently affect consumption across these households with high beverage consumption. Note that this still wouldn’t answer questions about the effect of reformulation or social marketing campaigns.*

2. The paper needs additional explanation for how these results can uniquely inform policymaking or practice.
The discussion should clearly state how policymakers or practitioners can use these results, if at all, to inform behavior change efforts. The discussion summarized the results well, but did not provide adequate explanation for why the results matter. It would strengthen the paper to provide this explanation, ideally for each main point covered in the discussion.

The main real-world implication offered is that these results reinforce that fiscal policies for obesity prevention should extend to sweet snacks. I don't disagree, but I wasn't sure why an LCA of beverage purchasing is an appropriate approach for understanding whether fiscal policies should extend to sweet snacks. Indeed, a better approach for understanding that question would seem to be the simulation modeling paper cited in reference 39.

*Response: This comment is very much related to the comment #1, with a specific focus on the concrete policy implication of this research. We have spelled out 3 policy implications in the revised version of the discussion (p29 paragraphs 3-5).*

In addition to these important limitations, I have also noted the following suggestions for each section of the paper.

Abstract
3. "…potentially compromising the effectiveness of these policies." I would strike. I very much agree we need to understand who is most affected to understand distributional effects of policies, but it's not clear that lacking this information undermines the policy's effects on behavior. (Won't the policy continue to raise prices and spur reformulation regardless of our understanding of who is affected?).

*Response: The statement was removed from the revised version of the manuscript.*

4. "…what kinds of fiscal policies might have the most impact on energy intake across this profile." I would strike. As noted above, this paper does not quantitatively examine what kinds of fiscal policies would have the most impact on energy intake. The discussion speculates on this question, but answering this question is not the core of the analysis and thus this should not be stated as an objective.

*Response: The statement was removed from the revised version of the manuscript.*

5. "households from the classes with higher total spend on SSBs had higher total energy purchases" - typo I think. Should be "total spending"?

*Response: This was modified avoid any confusion.*

6. "Fiscal policies to tackle SSB consumption may have differential effects depending on underlying household beverage purchase behaviours, which may negate or reinforce the policy objective to reduce sugar consumption and obesity." I don't disagree, but I do not think these results can speak to whether fiscal policies have differential effects on households, as no such effects were evaluated or simulated. I would replace this sentence with information on what these results uniquely tell us about policymaking.

*Response: The whole paragraph was edited to take into account this comments and comments from other reviewers.*

Introduction
7. "The two-tiered nature of the levy has encouraged the industry to reduce the sugar content of beverages, resulting in a decrease in the volume of sugars sold in beverages by 30% between 2015 and 2018." I don't disagree that the two-tiered nature of the levy may have encouraged reformulation; however, the construction of this sentence makes it seem like reformulation was the only driver of reduced beverage sugar purchased, with no role for consumer behavior change. Is that accurate? Please revise as needed.

*Response: Thank you for identifying an ambiguity in this sentence. The revised sentence is as follows:*

*“In addition to lowering demand for SSB, the two-tiered levy has encouraged the industry to reduce the sugar content of beverages, resulting in a decrease in the volume of sugars sold in beverages by 30% between 2015 and 2018”*

8. "However, understanding whether fiscal policies might be successful at reaching target populations and contribute to reducing SSB consumption and obesity prevalence remains a challenge." I think "contribute" should be "contributing" (same tense as "reaching"). Hard to follow this sentence.

*Response: The sentence was simplified to make it easier to follow. The whole section was substantially edited.*

9. I appreciated the brief explanation of LCA as a method to reduce analytic complexity. It would strengthen the paper to explain why LCA is the preferred approach for the earlier-stated research objective of understanding whether fiscal policies might be successful at reaching target populations. For example, why not conduct a simulation model disaggregated by key population group? Or examine cross-price elasticities within key groups?

*Response: in this paper, we did not assess whether policies might be successful at reaching high-risk groups. This is now clarified in the manuscript (throughout). Our main aim is to identify sub-groups which could be targets for interventions. We see LCA as a complementary approach to the modelling studies that the reviewer suggests. We do not think that one is more important or should be preferred over the other. Both approaches offer useful, yet different, information as inputs for policy. For example, as we already highlight above, elasticities could be calculated for pre-defined population groups based on hypothesised differences in responses, e.g. we know that those on lower incomes or socio-economic status are more price responsive. The LCA has however highlighted that among lower socio-economic status there are two distinct groups, one of which buys a lot of SSBs and some diet drinks (representing 18% of the sample) and the other buys a lot of diet drinks and almost no SSBs (16% of the sample). Cross elasticities for low socio-economic group (or for overweight/obese) would mask households with these two different behaviours. SSB policies which ultimately aim to lower obesity prevalence through reduced sugar intake would have no effect in the group that drinks predominantly diet beverages already. Thus the advantage of LCA is to be able to identify in a systematic way consumer groups that behave differently even though they may share same socio-economic/demographic characteristics that are associated with being at risk for obesity/overweight.*

*The reason for using LCA is now clarified in the introduction (paragraphs 3-4), in the methods (p10, ‘statistical analysis’ paragraph 1) and in the discussion (p29 paragraph 2).*

10. In the sentence about the benefits of purchase data, you might briefly note that these data do not capture food purchases from restaurants. (I know it's mentioned elsewhere, but this is a key limitation that needs to be stated alongside the key benefits of using purchase data).

*Response: This is now explicitly stated alongside.*

Methods
11. I would strike "representative" in the description of the Kantar data since you were not able to use sample weights.

*Response: The panel is representative of the GB households. Sample weights were indeed not included, but the household characteristics used to calculate the weights are included as covariates in the regression analyses. In other words, regression coefficients are unaffected by the non-inclusion of sample weights.*

12. Please include the % of products for which you needed to copy nutritional values across similar products. Likewise the % for which you needed to use the category average.

*Response: This information is specific for each nutrient variable in the dataset, so we decided to provide that information for energy (kcal) as an indication of the extent of imputation (p9 paragraph 2).*

13. I would suggest Figure S1 be in the main text to visually depict to readers the important point that the final sample is only ~1/5 of the starting sample.

Response: The figure is now included in the main text. Nonetheless, we wanted to stress that, compared to other studies, information on the total sample is not so relevant because of the way the Kantar FMCG works, and also because the focus of this paper is on regular beverage purchasers (i.e. subgroup analysis). The panel does not primarily aim to follow households over time, but products (in fact the final sample includes 27% of purchases while 20% of households). This is quite different from a traditional survey/trial the readers might be more familiar with.

14. The exclusion of households that don't regularly buy beverages was attributed to "modeling issues." This exclusion reduces the sample size by about 40% from what it would be including those HHs, so would be nice to have more understanding of why these HHs couldn't be included.

*Response: We thank the reviewer for identifying this ambiguity in our text. In the limitation section we indeed indicated that due to modelling issues we could include non-regular beverage purchasers. By this, we meant that although our primary focus was on regular beverage purchasers, we were unable to additionally investigate those who purchase beverages less regularly.*

*This issue was also raised by Reviewer 2 (comment #6). The rationale for excluding households that purchased less than 1L of beverage per week per household member was to have a better defined study population of regular beverage purchasers, who are those who are the policy targets of policies, such as the SDIL. Understanding the behaviours of irregular purchasers is not very interesting from a policy perspective, because their low level of consumption means that the policy is unlikely to have any health benefit for them. Furthermore, in the data analysis, irregular purchasers are likely to introduce a lot of noise (due to many zeros or very small values), which is likely to be well captured by the LCA because it is a data-driven method. Excluding them allowed to have a better focused picture of policy-relevant purchase patterns.*

*In addition, LCA, like any mixture model, is not very good at handling large datasets. More observations generally means more Latent Classes in the optimal solution, which eventually raises a lot of convergence issues in the models specifying many latent classes (which are then difficult to interpret!). This is because the function that needs to be optimised is very complex, and as a result, there is a high chance of finding local maxima in the estimation process.*

*In the revised version of the manuscript, we have clarified that our focus was on regular beverage purchasers (throughout introduction, discussion and conclusion + p8 paragraph 2). To avoid confusion, we removed the limitation, which mentioned that we could not compare regular and non-regular purchasers.*

15. I was curious about the exclusion of households with +/-3 SDs of beverage purchases. Why was this exclusion needed if you were using tertiles of purchases? Additionally, it was somewhat surprised that this exclusion resulted in a loss of about 20% of the sample. It would seem that much fewer than 20% of the sample would have values this extreme. But perhaps this restriction was applied to each beverage group, resulting in loss of different households for each beverage group? Some clarification would help. Additionally, given that this exclusion results in loss of quite a few households, additional justification should be provided as to why this exclusion is important and does not bias the results. For example, it would seem that excluding households at the low end (-3 SDs) on purchases would result in loss of key groups of HHs who are truly "low" purchasers of a given category. Also, I'm guessing that purchases are nearly always right skewed, so those at -3 SDs probably have 0 purchases of the products, which is not an implausible or unrealistic value for many households to have. (My household never buys SSBs, for example). Why does it make sense to exclude these HHs?

*Response: We thank the reviewer for this comment. In the initial version of the manuscript, only households with +3SD were excluded given that -3SD gave a negative value, and therefore no households with no purchases were excluded. These households with +3SD were excluded to avoid including panellists making mass purchases, which would fall outside household consumption (e.g. for a party), and would then create bias. This is why the restriction was applied on the beverage categories underlying used to define the beverages indicators (e.g. on beer and cider separately).*

*We appreciate the reviewer’s concern that this implies to a substantial drop in the number of observations and therefore could lead to some bias. In order to ensure that this was not the case, we have revised our definition of outliers in the revised version of the manuscript, and considered 3 samples:*

- *The initial restricted sample (+3SD) [not presented anymore]*
- *A slightly wider sample, applying the +3SD restriction to the beverage indicators themselves (e.g. beer & wine together) [sensitivity analysis]*
- *A wider sample, based on maximum plausible values for purchases on each beverage indicator (364L per year, i.e. 1/day on average), and in total (500L) [main result]*

*Results are consistent across these 3 sample restrictions and latent classes have similar interpretations and associations with demographic variables (with the difference that the optimal number of latent classes is 6 and/or 7 across the models). We decided to present the extended results in the main text and to include the results of the ‘slightly wider sample’ as sensitivity analyses (S5 Appendix). Results from the most restricted sample are not included, as the definition is more difficult to justify.*

16. In the measures section, would help to very briefly define SSBs. Same for fruit juices and milk-based juices. What are milk-based juices? May also help to clarify whether these would be subject to the UK SDIL.

*Response: Beverage categories are now clearly defined both in the main text (p9 paragraph 1) and in S2 Appendix. Upon verification, we found some misclassifications. In particular, some low-calories beverages were inadequately classified as SSBs. This has been corrected in the revised version of the manuscript. We have also adjusted the dilution rate to 1:4 and included it for diet squashes, which was not the case in the initial version of the manuscript.*

*In the revised version of the manuscript, we have ensured that the beverage classification is consistent with the following description. Note also that milk-based beverages were described by mistake as ‘milk-based juices’ in the initial version of the manuscript.*

*The classification is not based on the SDIL but rather on classification of beverages used in the UK, and in the literature more generally.*

|  | **Description** |
| --- | --- |
| **Beverages** |  |
| Sugar sweetened beverages (SSBs) | Sugary beverages, juices or squashes not included in the diet beverage category.  We assuming a dilution rate of 1:4 as used by the British Soft Drinks Association for concentrated squash/cordial |
| Diet beverages | Sugary beverages self-branded as ‘diet’, ‘low sugar’ or ‘No added sugar’, or with less than 1g of sugar/100ml;  Juices self-branded as ‘diet’, ‘low sugar’ or ‘No added sugar’, or with less than 1g of sugar/100ml;  Squashes self-branded as ‘diet’, ‘low sugar’ or ‘No added sugar’ and with less than 3g of sugar/100ml;  Flavoured water with less than 1g sugar/100mL  We assuming a dilution rate of 1:4 as used by the British Soft Drinks Association for concentrated squash/cordial. |
| Fruit juices & milk-based beverages | 100% Fruit juices and milk-based beverages (e.g. chocolate milk), excluding pure milk |
| Beer and cider | Any beer or cider |
| Wine | Wine, including sparkling wine and fortified wine |
| Bottled water | Still or sparkling bottled water |

17. Relatedly, I was curious that SSBs were not separated into higher and lower-sugar beverages that would be taxed differently. Can you explain the rationale for this decision?

*Response: We did not use the SDIL categories for two reasons. First, the low sugar category was very small before the introduction of reformulation and therefore for the data analysed. In a previous paper, we showed that in 2012-2013 the share of low sugar beverage (less than 8g/100ml) in total beverage expenditure was 2% against 15% for high sugar beverages (more than 8g) and 27% for diet beverages (Quirmbach et al.* [*https://jech.bmj.com/content/72/4/324*](https://jech.bmj.com/content/72/4/324)*). Second, the SDIL thresholds are not informative about the sugar purchased. A result of the SDIL has been the reformulation of product with an increase in the number of beverage with 4.5-4.9g of sugar/100ml. These won’t be subject to the levy but would still contain a substantial amount of sugar. We therefore used a definition of SSB as being beverage which are not ‘diet’, ‘no added sugar’ or low calories, which is more likely to inform about the underlying risks related to high sugar intake from beverages.*

18. Again, would help to explain what the "modeling issues" were that required examining beverage volume by tertile. Why not quartile? Quintile?

*Response: These modelling issues are now better explained (p9 end of paragraph 1). Tertiles were used to reduce the analytical complexity and ensure that the results of the LCA remain useable and interpretable (as noted by the reviewer in comment #28, even tertiles are already difficult to interpret). Tertiles were used following previous research, as noted in the manuscript (Joy et al. 2017* [*https://doi.org/10.1017/S1368980017000416*](https://doi.org/10.1017/S1368980017000416)*). In sensitivity analyses, we reproduced the analyses using quintile instead. Results are comparable and now presented in S5 Appendix.*

19. "Normal" BMI cutoffs - suggest instead referring to WHO. "Normal" cutoffs in Asia are not the same as in UK.

*Response: The sentence was edited accordingly.*

Results
20. "In addition to being classified in the Diverse class, households purchasing high volumes of SSBs were split…" This was confusing - made it seem like those in the Diverse class would further subdivided, when in fact I think you meant to refer to two other classes. The classes were not (based on my understanding) nested within one another, correct?

*Response: Thank you for spotting this confusing sentence. Indeed classes are not further divided. We have edited the sentence in the revised version of the manuscript.*

21. Throughout: Suggest using "person first" language. For example, several sentences refer to "obese respondents." I believe the recommended language is "Respondents with obesity" (as used elsewhere in the results).

*Response: The text was edited accordingly.*

22. How was the decision made to present the RRRs with the diverse class as the referent? I found it hard to know what to make of these results. Does the diverse class have some inherent meaning with regard to policymaking?

*Response: Thanks for this suggestion. RRRs are always difficult to present/interpret but these are the standard effect measure reported for this type of analysis (e.g. Mooney et al 2015* [*https://doi.org/10.1016/j.amepre.2015.02.015*](https://doi.org/10.1016/j.amepre.2015.02.015)*). As regularly done in this type of analysis, the reference group was chosen because it represents the biggest group and therefore the most typical type of household (and therefore reduces SEs). Furthermore it makes sense to choose this group as a reference category because it represents households with a diversity of beverage purchases. From a policy perspective, the ‘water’ class could make sense as well, however, due to the smaller sample size of the class, comparison against this group are generally less precise, which leads to wide uncertainty around the point estimates.*

*To ease interpretation, we have reported in Table 2 the % with high BMI (>25) and % with obesity (BMI>30) for each latent class. This shows more clearly than the RRRs that higher BMI is more prevalent in classes “SSB” and “Diet”.*

Discussion
(See above notes about highlighting what this paper uniquely adds to the literature).
23. The paragraph beginning "Our result suggests that fiscal policies to tackle SSB consumption may have differential effects…" I don't disagree with the sentiment here, but I wasn't sure how these results speak to that conclusion. The paper did not estimate or simulation how purchases would change among HHs in each beverage purchasing class, but rather describes current purchases. I would suggest revising this paragraph.

*Response: The sections in discussion regarding policy implications of the analysis have now been revised based on this reviewers and other reviewers’ comments (p29)*

24. "…proportional greater impact on households at highest risk of obesity." Again, not sure that this paper can speak to which households would be most impacted given behavior change was not modeled. Additionally, households are not at risk of obesity, individuals are - suggest revising.

*Response: The sentence was revised, as above explained.*

25. Limitations paragraph, "Patterns of beverage purchasing might different from those made to take-home." Couldn't quite follow this sentence.

*Response: Thank you for this comment. This is now corrected. It should have been “those made outside of home”.*

Misc/Throughout:
26. Given that this paper used purchase data, not dietary intake data, I would suggest avoiding referring to "diet" or "consumers" throughout. Instead, "purchases" and "purchasers." For example, "Households with less healthy diets overall" is inaccurate; diets were not assessed.

*Response: The reference to diet was removed. A consumer is defined as “a person who buys goods or services for their own use“, although it might also refer to what people eat. Given that the Kantar panel is defined as a consumer panel (Fast Moving Consumer Good), it appear difficult to avoid referring to consumers throughout the paper. We are nonetheless explicit throughout the manuscript that this study is not about self-reported dietary intake, but about beverage purchases.*

27. Tables did not fit onto page; formatting should be revised.

*Response: Tables in PLoS Medicine do not always fit onto one page (e.g.* [*https://doi.org/10.1371/journal.pmed.1002651*](https://doi.org/10.1371/journal.pmed.1002651)*) and this is not required in the instructions for author. We will revise upon editor request.*

28. Could the LCA results feasibly be shown as a figure? Might help make the results more digestible to readers.

*Response: We thank the reviewer for the suggestion. The table is now placed in S4 Appendix and it is replaced with a box plot by latent class of the distribution of each beverage type for the households assigned to the latent classes (Fig 2). This indeed gives a better visual interpretation of the latent classes.*

29. Figure S1 (flowchart) - Please add boxes for number excluded at each stage, not just number remaining. (Reader should not be asked to do the math on these; exclusion flowcharts should show both number retained and number excluded for each step).

*Response: Thank you. This was edited as suggested (Fig 1).*
